# Amerindian Ancestry Influences Genetic Susceptibility to Chronic Obstructive Pulmonary Disease

**DOI:** 10.3390/jpm10030093

**Published:** 2020-08-18

**Authors:** Roberto Díaz-Peña, Felix Boekstegers, Rafael S. Silva, Sergio Jaime, H. Dean Hosgood, Marc Miravitlles, Àlvar Agustí, Justo Lorenzo Bermejo, Jordi Olloquequi

**Affiliations:** 1 Laboratory of Cellular and Molecular Pathology, Instituto de Ciencias Biomédicas, Facultad de Ciencias de la Salud, Universidad Autónoma de Chile, Talca 3460000, Chile; roberdp78@gmail.com; 2 Liquid Biopsy Analysis Unit, Oncomet, Health Research Institute of Santiago (IDIS), 15706 Santiago de Compostela, Spain; 3 Statistical Genetics Group, Institute of Medical Biometry and Informatics, University of Heidelberg, 69126 Heidelberg, Germany; boekstegers@imbi.uni-heidelberg.de (F.B.); lorenzo@imbi.uni-heidelberg.de (J.L.B.); 4 Unidad Respiratorio, Centro de Diagnóstico Terapéutico, Hospital Regional de Talca, Talca 3460000, Chile; rafaelsilvao@gmail.com (R.S.S.); sergiomet@gmail.com (S.J.); 5 Department of Epidemiology and Population Health, Albert Einstein College of Medicine, Bronx, NY 10461, USA; dean.hosgood@einsteinmed.org; 6 Pneumology Department, Hospital Universitari Vall d’Hebron/Vall d’Hebron Institut de Recerca (VHIR), CIBER Enfermedades Respiratorias (CIBERES), 08035 Barcelona, Spain; marcm@separ.es; 7 Respiratory Institute, Hospital Clínic, Institut d’Investigacions Biomèdiques August Pi i Sunyer (IDIBAPS), Universitat de Barcelona, CIBER Enfermedades Respiratorias (CIBERES), 08036 Barcelona, Spain; aagusti@clinic.cat

**Keywords:** chronic obstructive pulmonary disease (COPD), ancestry, autoimmunity, genome wide association studies (GWAS), personalized medicine, Hispanic paradox, immune dysfunction

## Abstract

The contribution of genetic ancestry on chronic obstructive pulmonary disease (COPD) predisposition remains unclear. To explore this relationship, we analyzed the associations between 754,159 single nucleotide polymorphisms (SNPs) and risk of COPD (*n* = 214 cases, 193 healthy controls) in Talca, Chile, considering the genetic ancestry and established risk factors. The proportion of Mapuche ancestry (PMA) was based on a panel of 45 Mapuche reference individuals. Five *PRDM15* SNPs and two *PPP1R12B* SNPs were associate with COPD risk *(p* = 0.05 to 5 × 10^−4^) in those individuals with lower PMA. Based on linkage disequilibrium and sliding window analyses, an adjacent *PRDM15* SNPs were associated with COPD risk in the lower PMA group *(p* = 10^−3^ to 3.77 × 10^−8^). Our study is the first to report an association between *PPP1R12B* and COPD risk, as well as effect modification between ethnicity and *PRDM15* SNPs in determining COPD risk. Our results are biologically plausible given that *PPP1R12B* and *PRDM15* are involved in immune dysfunction and autoimmunity, providing mechanistic evidence for COPD pathogenesis and highlighting the importance to conduct more genome wide association studies (GWAS) in admixed populations with Amerindian descent.

## 1. Introduction

Chronic obstructive pulmonary disease (COPD) is a common disorder characterized by a persistent and progressive airflow limitation that is associated with an enhanced chronic inflammatory response in the airways and lungs to noxious particles or gases, particularly those attributed to cigarette and biomass smoke [1,2]. COPD is the fourth leading cause of death worldwide and it is projected to become the third by 2020 [1,3]. According to the World Health Organization, approximately 3 million people in the world die as a consequence of COPD every year, and the disease burden is increasing in Latin America [4]. Latin American countries experience a COPD prevalence of 13.4%, with in-hospital mortality rate ranging from 6.7% to 29.5% [5]. The prevalence of COPD increases steeply with age, with the highest prevalence among those people over 60 years [6]. Considering the high rates of tobacco use and biomass burning in several Latin American countries [7,8], COPD may become an even greater health problem in Latin America than previously hypothesized. In Chile, for example, respiratory diseases are the third most common cause of death, with COPD accounting for 22% of the deaths and being the second cause of decease [9].

In general, Latin Americans have been considered homogenous in most genome-wide association studies (GWAS), ignoring the degree of admixture variation between Latin American countries according to the major ancestry population component. However, both Latin Americans and Hispanics/Latinos in other parts of the world are characterized by broad ethnic diversity [10], with different admixture proportions of Amerindian, European, and African ancestry between and within countries, attributed to distinct populations of origin and historic characteristics of European settlement and slave-trading [11,12,13,14]. In Chile, for example, Amerindian ancestry proportions are higher in the north and the south, whereas European ancestry proportions are highest in central regions [15,16]. Conversely, the contribution of African ancestry proportions decreases from north to south [15,16].

Most genetic association studies of COPD have been in Caucasian, African, and Asian populations, often with controversial results depending on the population studied [17], suggesting that COPD susceptibility may depend on diverse ethnic gene–environment interactions. To date, over 97 independent genetic loci have been associated with the lung parameters defining COPD—forced expiratory volume in one second (FEV_1_), forced vital capacity (FVC), or the FEV_1_/FVC ratios—and with COPD risk [18,19]. Notably, single nucleotide polymorphisms (SNPs) near or in *HHIP*, *FAM13A*, *ADAM19*, and *CHRNA3/5* have been frequently associated with COPD risk [18,20,21,22,23]. Regarding lung function or COPD-related phenotypes, only four GWAS have been focused on Hispanic/Latino populations, reporting novel loci (in or near the genes *KLHL7/NUPL2*, *DLG2*, *PDZD2*, and *PRDM15*), as well as others previously identified in non-Hispanic populations [24,25,26,27]. Therefore, further genetic studies on non-Caucasian populations are required in order to understand whether ancestry plays a key role in the pathogenesis of COPD [28,29].

The present study, conducted in El Maule, a rural region of Chile, explored the risk of COPD associated with established risk factors and identified susceptibility variants both independently and in conjunction with genetic ancestry.

## 2. Materials and Methods

### 2.1. Study Population

The El Maule Region, located about 250 km south of Santiago, is one of the most rural counties in Chile. El Maule experiences some of the highest levels of air pollution attributed to biomass burning in Chile. Moreover, according to the Chilean Ministry of Health, El Maule shows high mortality rates attributed to COPD, pneumonia, and respiratory system malignancies (Supplementary Figure S1). For this study, we recruited 214 Maulean patients with COPD at the respiratory service of the Hospital Regional de Talca, where they attended to undergo diagnostic tests after suspected COPD or for COPD monitoring visits. Control subjects (*n* = 193) with no record of any specific illness were enrolled in parallel at the same Hospital through a volunteer recruitment program. The Ethics Committees of the Maulean Health Service and Universidad Autónoma de Chile approved the study and all subjects provided written informed consent (approval code: 063-15).

The Global Initiative for Chronic Obstructive Lung Disease (GOLD) criteria [30] were used for the diagnostic evaluation of subjects. Standard clinical information and medical history was collected. Pulmonary function—including measurements of forced expiratory volume in 1 second (FEV_1_), forced vital capacity (FVC), and carbon monoxide diffusing capacity of the lung (DL_CO_)—was assessed in all subjects using standard procedures [31] and equipment (Masterlab; Jaeger, Würzburg, Germany). Oxygen saturation was also measured by pulse-oximetry (Ohmeda TuffSat, Soma Technology, Bloomfield, Connecticut, USA). Body mass index (BMI) was calculated using the current weight and height of each study participant. Dyspnea was determined using the modified Medical Research Council scale (mMRC) and exercise capacity was determined with the distance walked in 6 minutes test (6MWT). Health-related quality of life and symptom burden in patients with COPD was quantified using the COPD assessment test (CAT). A Body mass, airflow Obstruction, Dyspnea, and Exercise (BODE) score was assigned to each COPD patient. Cigarette smoking history was measured by pack-years and cumulative exposure to biomass smoke (hour-years) was calculated as previously described [32]. 

Finally, patients with asthma–COPD overlap syndrome were identified and excluded if they had a history of asthma, rhinitis, or any extra-pulmonary disease affecting lung function, and with positive bronchodilator test, FEV_1_ increasing by ≥12% and 200 mL. Participants with a COPD exacerbation or hospitalization record during the previous two months were also excluded, to ensure that COPD patients were stable.

### 2.2. Genotyping, Imputation and Ancestry Estimation

When visiting the respiratory service, 5 mL of blood was collected from each participant and stored in plastic vacutainer tubes containing ethylenediaminetetraacetic acid (EDTA). DNA from peripheral blood cells was extracted using the GeneJET Genomic DNA purification kit #K0722 (Thermo Fisher Scientific, Waltham, Massachusetts, USA), following the manufacturer’s instructions. Samples were stored at −80 °C until genotyped using the Illumina Global Screening Array [27].

We investigated 89 SNPs (Supplementary Table S1), that were previously associated with COPD risk at genome-wide significance in large European GWAS [21,33,34,35]. Since 46 of these SNPs were not included in the array, we imputed them using the IMPUTE2 software version 2.3.2 with version 3 of the 1000 Genomes Project data as the reference set, as previously described [36,37]. We used the ADMIXTURE software, version 1.3, for supervised estimation of individual European, African, Mapuche, and Aymara ancestry components [38]. The 1000 Genome project contributed individual surrogates of European and African ancestry: 99 Utah residents with northern and western European ancestry (CEU), 107 individuals from Iberian populations in Spain (IBS), and 108 Yorubans in Ibadan, Nigeria (YRI) [37]. Lorenzo et al. had identified nine Mapuche and nine Aymara in the Chilean study population of the Consortium for the Analysis of the Diversity and Evolution of Latin America [39]. The Mapuche reference individuals were complemented by four Huilliche from Reich et al. and 32 Huilliche–Pehuenche from Lindo et al., and the Aymara reference individuals were complemented by 22 Aymara and 40 Quechua from Reich et al. [40,41]. Finally, we assessed the distribution of all the SNPs previously evaluated in a part of this Chilean cohort [27] considering the genetic ancestry.

To explore linkage disequilibrium (LD) pattern in the *PRDM15* gene, we accessed the 1000 Genomes genetic data of 629 individuals from different ethnic backgrounds via FTP download (available at ftp://ftp-trace.ncbi.nih.gov/1000genomes/ftp/release/20100804/ALL.2of4intersection.20100804.genotypes.vcf.gz), including 261 Europeans (EUR), 177 East Asians (EAS), 169 Africans (AFR), and 22 admixed Americans (AMR).

### 2.3. Statistical Analyses

SNPs that met the quality control (QC) criteria of a minor allele frequency (MAF) > 0.01, missing call rate < 0.2, and/or Hardy–Weinberg equilibrium (HWE) *p* > 0.001 were considered for inclusion in the association analyses. Of the 89 SNPs, 80 passed QC and were included in the analysis (Supplementary Table S1). When we analyzed all the SNPs previously evaluated (*n* = 455,564) [27], allele frequencies were compared between COPD patients and controls by χ^2^ test, and odds ratios (OR) with 95% confidence intervals (95% CI) were calculated using PLINK software (v1.07) [42]. Sliding window analyses, assessing the frequency of composite genotypes of a fixed number of contiguous SNPs, were tested for association analyses by χ^2^ test with PLINK software [42]. Haplotype analysis was performed with Haploview version 4.1 software [43], using the CI method to perform LD assessment and define haplotype blocks. Associations between identified COPD susceptibility genetic markers and potential interactions with genetic ancestry were tested using logistic regression, considering COPD diagnosis as response variable. An additive model was assumed for individual genotypes and ancestry proportions were included as continuous covariates. The SNPAssoc package of R was used to investigate the associations between COPD risk and the combined effects of SNPs associated with COPD risk, established COPD risk factors and individual ancestry proportions, using multiple logistic regression models [44].

## 3. Results

### 3.1. Demographic and Clinical Findings

**Figure 1:**
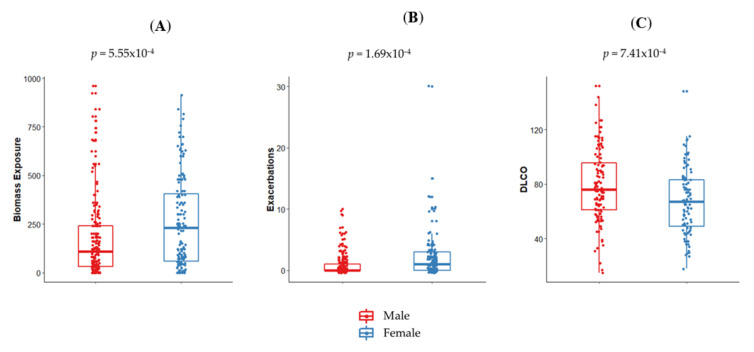
Sex differences in clinical and exposure parameters in chronic obstructive pulmonary disease (COPD) patients. Female COPD patients showed an increased exposure to in-home biomass smoke (**A**) and exacerbations in the previous year (**B**), whereas males exhibited a better carbon monoxide diffusing capacity of the lung (DL_CO_) (**C**).

In our population, COPD patients and controls were of similar age (*p* = 0.69) but differed in sex proportions (*p* = 7.3 × 10^−6^), and controls showed a significantly higher average of schooling years (*p* = 2.2 × 10^−15^; Table 1). Interestingly, BMI was lower in COPD patients (*p* = 1.79 × 10^−5^), although both groups were generally overweight (Table 1). COPD patients exhibited higher amounts of cigarette smoking and cumulative exposure to biomass smoke than controls (*p* = 2.45 × 10^−12^ and *p* = 3.98 × 10^−4^, respectively). COPD patients also had reduced FEV_1_, FEV_1_/FVC, DL_CO_, oxygen saturation, and 6MWT compared to controls (*p* = 2.2 × 10^−15^ to *p* = 2.15 × 10^−7^). Most COPD patients were GOLD stage 2 (FEV_1_ between 50% and 79% of predicted). Female patients showed a higher exposure to biomass smoke and an increased number of exacerbations in the previous year (*p* = 5.5 × 10^−4^ and *p* = 1.69 × 10^−4^, respectively; Figure 1A,B). In contrast, male patients exhibited an increased DL_CO_ (*p* = 7.41 × 10^−4^; Figure 1C).

### 3.2. Analysis of Population Structure 

**Figure 2:**
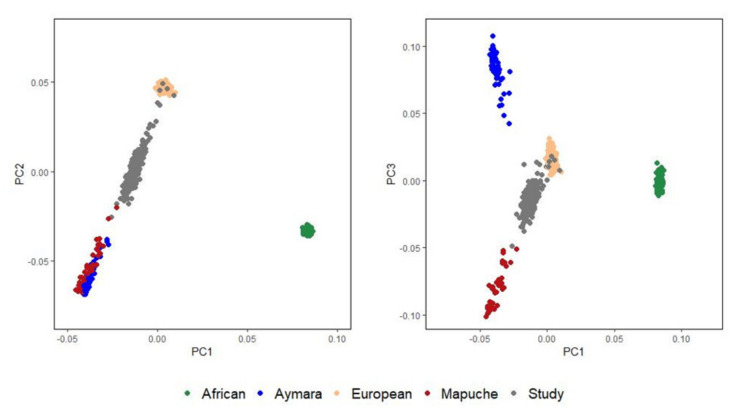
Genetic principal component analyses of study individuals and European, African, Mapuche, and Aymara reference individuals.

We conducted genetic principal component analyses using the EIGENSTRAT function available at popgen.dk/software/index.php/Rscripts [45]. The first principal component (PC1) distinguished Africans from non-Africans and the second principal component (PC2) separated European and Native American ancestry components. The third principal component (PC3) separated the Mapuche and Aymara Native American subcomponents. Major influences of the European and Mapuche ancestries in the study population were revealed (Figure 2). In our cohort, the average Aymara proportion was 6.48%, whereas the average Mapuche percentage was 35.24%. The average European and African proportions were 55.61% and 1.59%, respectively. COPD patients and controls were similar with respect to ancestry proportions (Table 1).

### 3.3. Influence of Genetic Ancestry on the Association of SNPs and COPD

Four of our 80 a priori SNPs were associated with COPD risk: rs626750 (OR 0.57, 95% CI 0.37–0.87, *p* = 0.01), rs9599114 (OR 0.75, 95% CI 0.57–0.99, *p* = 0.05), rs7181486 (OR 1.40, 95% CI 1.01–1.93, *p* = 0.04), and rs8034191 (OR 1.44, 95% CI 1.04–1.99, *p* = 0.03). Only rs207675 showed suggestive association after accounting for covariates (OR 0.50, 95% CI 0.28–0.90, *p* = 0.02; Table 2).

When exploring all variants of the Illumina Global Screening Array, rs12741415 (*PPP1R12B* gene), rs116062217 (*PPP1R12B* gene), rs1054761 (*PRDM15* gene), rs4075967 (*PRDM15* gene), and rs4862451 (intergenic) were associated to COPD risk (*p* < 10^−5^; Supplementary Table S2 and Supplementary Figure S2). The top four hits map to *PPP1R12B* and *PRDM15*, on chromosomes 1 and 21, respectively. In order to explore the influence of the proportion of Mapuche ancestry (PMA) on the association between the SNPs markers and COPD, we stratified the analysis by PMA levels expressed in two categories (low vs. high PMA) based on the median value of this variable (35%). The association between the *PRDM15* variants and COPD was present in those individuals within the lower PMA group (Table 3). In addition, *PRDM15* rs1054761 (OR = 0.23, *p* = 0.02), rs2236696 (OR = 10.41, *p* = 1.00 × 10^−3^), rs4075967 (OR = 0.22, *p* = 0.05), rs4075970 (OR = 6.76, *p* = 8.25 × 10^−4^) and rs28360603 (OR = 7.55, *p* = 5.41 × 10^−4^), were associated with risk in those with lower PMA after adjusting for age, sex, smoking status, biomass exposure, scholarship, and body mass index (Table 3).

**Figure 3:**
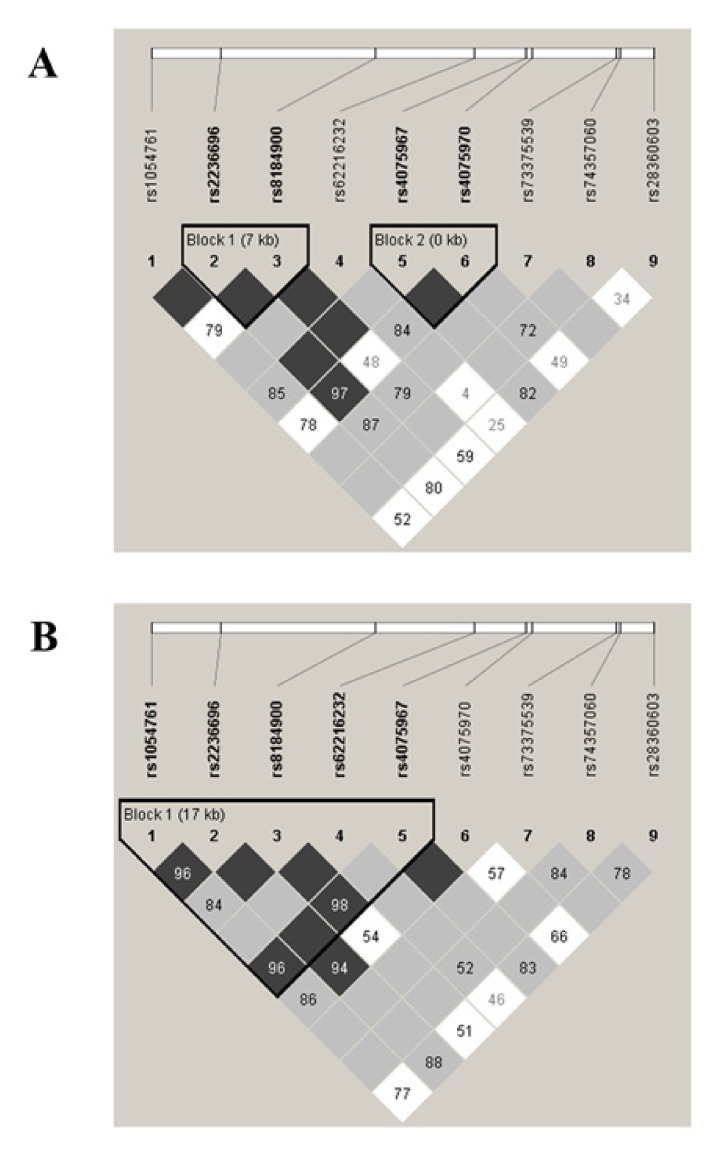
Linkage disequilibrium plots around the *PRDM15* gene in the lower (**A**) and higher (**B**) proportion of Mapuche ancestry (PMA) groups. Squares represent the linkage disequilibrium (D’) value between every two single nucleotide polymorphisms: the darker the square, the higher D’ value. The five triangular shapes surrounding these markers indicate the haplotype blocks that were defined using the confidence interval method.

The sliding window omnibus test revealed several SNP blocks associated with COPD in the lower PMA group (Table 4). Hence, the *p*-values obtained for the sliding window test of a region comprising *PRDM15* gene (Chr21: 43.229.099 to 43.256.172) were lower than 10^−6^. We also performed case–control regression analysis based on haplotype block reconstruction, and detailed haplotype block information and the LD plot around the *PRDM15* gene are shown in Figure 3. In the lower PMA group, *PRDM15* was divided into two LD blocks, both with two SNPs (Figure 3A), showing association with COPD (positions 43,221,826 through 43,229,099, CG (*p* = 7.40 × 10^−6^, *p*-value using 10,000 permutations <10^−3^); and positions 43,236,176 through 43,236,481, AG (*p* = 1.01 × 10^−7^, *p*-value using 10,000 permutations <10^−4^]). Differences were also found in the LD pattern among the higher PMA group (Figure 3B). No differences were observed around *PRDM15* gene. When data across the four superpopulations (EUR, EAS, AMR, and AFR) were compared, high inter-ethnic differences were found in the LD patterns and haplotype blocks compared to our control population (Supplementary Figure S3).

## 4. Discussion

The present study is the first to report the contribution of established risk factors, genetic ancestry, and SNPs associated to COPD risk in a Latin American population, characterized by a large admixture of Amerindian ancestry.

Our analysis highlighted the unique demographic and clinical characteristics of our study population. Almost a fifth of all COPD patients were never-smokers, whereas most experienced a high exposure to biomass smoke. These exposure scenarios, coupled with the low educational level, reflect the rural nature of our population. Maulean COPD patients were also overweight, coinciding with studies in Chile and elsewhere [46,47]. Interestingly, females were more exposed to indoor biomass smoke than males, as have been reported in other studies performed in middle-income or developing countries [2]. Considering the advanced age of the participants, this probably reflects a sociocultural effect, since decades ago Chilean women followed a different lifestyle than men, remaining at unventilated homes during long periods, cooking, or being near woodstoves. The higher exposure to biomass smoke could also explain the fact that female COPD participants showed a decreased DL_CO_ than men. Indeed, it has been reported that biomass smoke-related COPD is associated with milder emphysema and DL_CO_ reduction than tobacco-related COPD [48]. Our results are similar to recent reports of higher COPD exacerbation frequency among women [49,50], which may reflect the higher hyperresponsiveness prevalence reported in females [51]. Further, exposure to household biomass smoke pollutants, such as PM_2.5_ and NO_2_, have been associated with increased risk of COPD exacerbations [52].

We found that Amerindian ancestry-specific alleles in *PRDM15* may confer a protection against COPD. Our results are biologically plausible given that *PRDM15* has been shown to play a key role in regulating WNT and MAPK-ERK signaling [53] through regulation of the MAPK-ERK signaling cascade. In addition, rs12741415 and rs116062217, located in the *PPP1R12B*, were associated with COPD susceptibility in our population. The protein encoded by the gene *PPP1R12B*, a protein phosphatase 1 regulatory subunit 12B, is a component of myosin phosphatase complex, which regulates contractile processes in muscular and other cells [54]. Interestingly, Freĭdin and coworkers postulated *PPP1R12B* as a candidate gene for childhood asthma [55], but these data have not been replicated in other populations. It is also noteworthy that both *PRDM15* and *PPP1R12B* have been related to immune dysfunction and/or autoimmunity, mechanisms that are thought to play a key role in COPD pathogenesis [56]. Hence, PRDM15 is overexpressed in B-cell dysregulation [57,58], a characteristic feature of COPD [59], whereas *PPP1R12B* and its “M20” transcript are higher in patients with celiac disease autoimmunity [60]. Although further research is needed to deeper understand the potential contribution of *PRDM15* and *PPP1R12B* to COPD, resequencing *PRDM15* and *PPP1R12B* may clarify the nature of the genetic variation and develop a functional study of their protein products in this complex disease.

The results of the present study suggest that the risk allele frequencies of some SNP may vary in Europeans and Chileans. Given our findings, and that allele frequencies of disease-associated SNPs may vary by ethnic group due to genetic drift or selection [61], it is important to account for ancestry to avoid false associations. The results of the present study showed little replication of previously reported genetic associations with COPD, with only 10.1% of known COPD loci from GWAS in populations of European origin showing a suggestive association in our Latin American cohort (*p* < 0.1). For example, our population exhibited as smaller allele frequency of rs207675-C (our control population = 0.48) relative to European populations (https://www.internationalgenome.org/; 1000 genomes project (EUR) = 0.64). Assuming our findings are replicated in another population, they will have implications for the implementation of personalized medicine which most often focuses on specific biological, genetic, and clinical characteristics of an individual. Our results suggest that there are other factors that provide additional complexity that need to be considered, such as ethnicity, life-style differences, and environmental factors. In this context, there is a relevant gap to overcome between European and non-European target populations, which is necessary for the implementation of the personalized medicine in all populations [62].

While these results are intriguing, we must acknowledge that our study is limited by the sample size and lack of replication. However, due to the heterogeneity of its genetic structure, getting ideal ancestral populations in Latin Americans is more difficult than in other admixed populations, which confers relevance to what is found in our population [63]. Moreover, since approximately 79% of all GWAS participants are Caucasians, and the fraction of non-European individuals in GWAS has stagnated or declined since late 2014 [64], our preliminary results give a valuable idea of the heterogeneity in genetic susceptibility to COPD in diverse ethnic groups.

In summary, the present study shows that *PRDM15* and *PPP1R12B* are associated with COPD risk in a Latin American population. Moreover, the association of *PRDM15* variants conferring genetic protection to COPD is higher in patients with more Amerindian ancestry. Although the relative low sample size and the lack of replication limit the implications of the present study, our results stress the need to conduct more GWAS in admixed populations, such as those with Amerindian descent.

## Figures and Tables

**Table 1:** Demographic, clinical, and genetic ancestry data of the study population.

-	Control Subjects *n* = 193	COPD Patients *n* = 214
Sex, Male/Female	60/133	121/93
Age, years	68.66 ± 3.25	70.97 ± 4.69
Smoking history, pack-years	7.75 ± 3.25	30.47 ± 14.82 *
Current Smoker	31 (16.06%)	33 (15.42%)
Former smoker	71 (36.79%)	145 (67.76%)
Never smoker	91 (47.15%)	36 (16.82%)
Biomass exposure, hour-years	96.87 ± 32.57	225.62 ± 54.28 *
Scholarship, years	14.33 ± 2.57	7.21 ± 3.98 *
BMI, kg/m^2^	29.45 ± 5.02	26.96 ± 5.02 *
Exacerbations in the previous year	––	1.37 ± 1.50
FEV_1_, % predicted	108.84 ± 18.40	61.47 ± 24.56 *
FEV_1_/FVC, %	83.00 ± 6.27	58.25 ± 10.48 *
DL_CO_, % predicted	87.43 ± 24.48	72.33 ± 25.13 *
Oxygen Saturation, %	96.14 ± 2.34	92.36 ± 4.76 *
6MW, meters	462.95 ± 87.82	351.50 ± 155.61 *
mMRC	––	2.28 ± 1.39
CAT	––	14.94 ± 8.46
BODE	––	3.18 ± 2.74
European ancestry, %	55.93 ± 6.89	56.32 ± 10.27
African ancestry, %	1.73 ± 1.00	1.73 ± 1.12
Mapuche ancestry, %	35.39 ± 6.71	35.11 ± 8.54
Aymara ancestry, %	6.96 ±.45	6.73 ± 3.52

Data presented as mean ± standard deviation, unless otherwise indicated. Definition of abbreviations: BMI: body mass index; FEV_1_: forced expiratory volume in 1 second; FVC: forced vital capacity; DL_CO_: carbon monoxide diffusing capacity; 6MW: 6 minutes walking test; mMRC: modified Medical Research Council scale; CAT: Chronic obstructive pulmonary disease (COPD) assessment test; BODE: Body mass, airflow Obstruction, Dyspnea, and Exercise index. * Different from control subjects (*p* < 0.05).

**Table 2:** Common variants associated with COPD susceptibility in Caucasian and Chilean population (only SNPs with *p* ≤ 0.05 are shown).

	CAUCASIAN			CHILEAN							
SNP	SNP Risk Allele	Risk Allele Frequency	OR	A1	MAF Controls	MAF COPD Cases	OR1	*p*	*OR2	95% CI	*p*
rs626750	T	0.17	0.74	T	0.15	0.09	0.57	0.01	0.50	0.28–0.90	0.02
rs8034191	C	0.33	1.40	C	0.21	0.27	1.44	0.03	1.33	0.86–2.07	0.20
rs7181486	C	0.39	1.32	T	0.21	0.27	1.40	0.04	1.39	0.90–2.15	0.14
rs9599114	C	0.42	0.11	C	0.47	0.40	0.75	0.05	0.77	0.53–1.13	0.18

SNP: single nucleotide polymorphism; A1: minor allele nucleotide; MAF: minor allele frequency; OR: odds ratio; *p*: *p*-value; CI: confidence interval. *OR adjusted for sex, smoking status, biomass exposure, scholarship, and body mass index.

**Table 3:** Association between *PRMD15* SNPs and COPD risk in participants with low and high PMA (proportion of Mapuche ancestry, <35% vs. ≥35%).

**Low PMA**							
**SNP**	**A1**	**OR1**	**95%CI**	***p***	*OR2	**95%CI**	***p***
rs1054761	T	0.31	0.18–0.51	7.24 × 10^–7^	0.23	0.09–0.89	0.02
rs2236696	T	2.45	1.5–3.99	1.89 × 10^–4^	10.41	1.99–54.59	1.00 × 10^−3^
rs8184900	G	0.4	0.26–0.62	9.99 × 10^–6^	0.40	0.11–1.49	0.16
rs4075967	A	0.27	0.16–0.46	9.46 × 10^–8^	0.22	0.04–1.12	0.05
rs4075970	A	2.43	1.57–3.78	3.32 × 10^–5^	6.76	1.84–24.84	8.25 × 10^−4^
rs28360603	A	2.26	1.46–3.48	1.24 × 10^–4^	7.55	2–28.54	5.41 × 10^−4^
rs7275618	C	1.79	1.18–2.71	4.75 × 10^–3^	2.04	0.57–7.30	0.25
rs35109371	C	1.87	1.24–2.82	2.16 × 10^–3^	2.02	0.56–7.26	0.27
**High PMA**							
**SNP**	**A1**	**OR1**	**95%CI**	***p***	*OR2	**95%CI**	***p***
rs1054761	T	0.63	0.41–0.96	0.03	0.41	0.13–1.25	0.10
rs2236696	T	1.02	0.7–1.5	0.92	2.34	0.80–6.92	0.11
rs8184900	G	0.73	0.5–1.08	0.12	0.58	0.29–1.16	0.12
rs4075967	A	0.73	0.47–1.13	0.15	0.45	0.15–1.40	0.15
rs4075970	A	0.98	0.68–1.43	0.93	2.08	0.77–5.64	0.13
rs28360603	A	1.22	0.82–1.79	0.32	1.04	0.34–3.17	0.94
rs7275618	C	1.18	0.77–1.83	0.44	0.81	0.22–3.01	0.75
rs35109371	C	1.32	0.88–1.96	0.17	1.08	0.31–3.71	0.90

SNP: single nucleotide polymorphism; A1: minor allele nucleotide; OR: odds ratio; *p*: *p*-value; CI: confidence interval; PMA: proportion of Mapuche ancestry.*OR adjusted for sex, smoking status, biomass exposure, scholarship, and body mass index.

**Table 4:** Association analyses of sliding windows of 2–10 single nucleotide polymorphisms each, using chi-square statistics within PLINK software (only *p*-values <10^−6^ are shown).

SNP Markers	*p*-Value
rs4075967|rs4075970	1.01 × 10^−7^
rs1054761|rs2236696|rs8184900	9.53 × 10^−7^
rs2236696|rs8184900|rs62216232	3.77 × 10^−8^
rs62216232|rs4075967|rs4075970	1.43 × 10^−7^
rs2236696|rs8184900|rs62216232|rs4075967	1.01 × 10^−7^
rs8184900|rs62216232|rs4075967|rs4075970	1.16 × 10^−7^
rs62216232|rs4075967|rs4075970|rs73375539	1.44 × 10^−7^
rs4075967|rs4075970|rs73375539|rs74357060	1.42 × 10^−7^
rs2236696|rs8184900|rs62216232|rs4075967|rs4075970	9.48 × 10^−8^
rs8184900|rs62216232|rs4075967|rs4075970|rs73375539	8.36 × 10^−8^
rs62216232|rs4075967|rs4075970|rs73375539|rs74357060	1.09 × 10^−7^
rs4075967|rs4075970|rs73375539|rs74357060|rs28360603	4.38 × 10^−7^
rs2236696|rs8184900|rs62216232|rs4075967|rs4075970|rs73375539	9.52 × 10^−8^
rs8184900|rs62216232|rs4075967|rs4075970|rs73375539|rs74357060	5.81 × 10^−8^
rs62216232|rs4075967|rs4075970|rs73375539|rs74357060|rs28360603	5.80 × 10^−7^
rs2236696|rs8184900|rs62216232|rs4075967|rs4075970|rs73375539|rs74357060	1.61 × 10^−7^
rs8184900|rs62216232|rs4075967|rs4075970|rs73375539|rs74357060|rs28360603	4.78 × 10^−7^
rs62216232|rs4075967|rs4075970|rs73375539|rs74357060|rs28360603|rs28708536	4.29 × 10^−7^
rs2236696|rs8184900|rs62216232|rs4075967|rs4075970|rs73375539|rs74357060| rs28360603	6.39 × 10^−7^
rs8184900|rs62216232|rs4075967|rs4075970|rs73375539|rs74357060|rs28360603| rs28708536	3.76 × 10^−7^
rs62216232|rs4075967|rs4075970|rs73375539|rs74357060|rs28360603|rs28708536| rs76291974	2.28 × 10^−7^
rs8184900|rs62216232|rs4075967|rs4075970|rs73375539|rs74357060|rs28360603| rs28708536|rs76291974	1.79 × 10^−7^
rs62216232|rs4075967|rs4075970|rs73375539|rs74357060|rs28360603|rs28708536| rs76291974|rs62214694	2.86 × 10^−7^
rs8184900|rs62216232|rs4075967|rs4075970|rs73375539|rs74357060|rs28360603| rs28708536|rs76291974|rs62214694	2.17 × 10^−7^
rs62216232|rs4075967|rs4075970|rs73375539|rs74357060|rs28360603|rs28708536| rs76291974|rs62214694|rs17766525	2.66 × 10^−7^

## Supplementary Materials

The following are available online at https://www.mdpi.com/2075-4426/10/3/93/s1: Supplementary Figure S1: Analysis of mortality rate in Chile; Supplementary Table S1: Eighty-nine common single nucleotide polymorphisms (SNPs) included in the study from previous genome wide association studies (GWAS) in populations of diverse ethnic background; Supplementary Figure S2: Single-marker allelic association results; Supplementary Table S2: Single-SNP allelic association analysis (*p* < 10^−5^); Supplementary Figure S3: Linkage disequilibrium plots around the *PRDM15* gene.
