# Supplementary material for: Amerindian Ancestry Influences Genetic Susceptibility to Chronic Obstructive Pulmonary Disease
Source: J Pers Med. 2020 Aug 18;10(3):93. doi: 10.3390/jpm10030093 (PMC7565405; doi:10.3390/jpm10030093)
Supplement: Supplementary file 1 [file jpm-10-00093-s001.pdf]

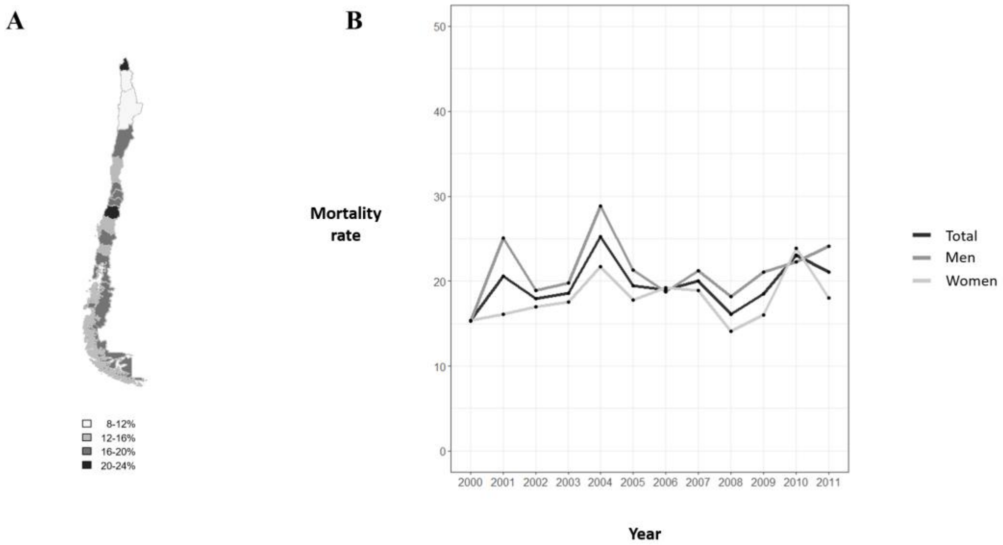

Figure S1. Analysis of mortality rate in Chile. A. Map with regional mortality rates due to COPD in Chile (2011); B. Mortality rate due to COPD in Maule region from 2001-2011.

Table S1. Eighty-nine common SNPs included in the study from previous GWAS in populations of diverse ethnic background.

ID	SNP	Position**	Region	Gene/nearest gene(s)	Quality Control
**1**	rs114216682*	53871085	1p32.3	FLJ40434	pass
**2**	rs17111652*	55590465	1p32.3	USP24	pass
**3**	rs4846480	218598469	1q41	TGFB2	pass
**4**	rs10429950*	218624533	1q41	TGFB2	pass
**5**	rs1737890*	31042595	20q11.21	NOL4L	pass
**6**	rs12692398	10200316	2p25.1	CYS1	pass
**7**	rs16825267*	229569919	2q36.3	PID1	pass
**8**	rs1529672	25520582	3p24.2	RARB	pass
**9**	rs41526344*	2985142	3p26.2	CNTN4	pass
**10**	rs2955083*	127961178	3q21.3	EEFSEC	pass
**11**	rs546764*	138294336	3q22.3	CEP70	pass
**12**	rs10023464*	69659738	4q13.2	UGT2B10	pass
**13**	rs4416442	89866713	4q22.1	FAM13A	pass
**14**	rs2869966	89869078	4q22.1	FAM13A	pass
**15**	rs2869967	89869332	4q22.1	FAM13A	pass
**16**	rs7674369	89872176	4q22.1	FAM13A	pass
**17**	rs7671167	89883979	4q22.1	FAM13A	pass
**18**	rs6837671*	89873092	4q22.1	FAM13A	pass
**19**	rs1964516*	89875909	4q22.1	FAM13A	pass
**20**	rs2047409*	106137033	4q24	TET2	HWE p<0.05
**21**	rs17035917*	106520742	4q24	ARHGEF38	pass
**22**	rs11727735*	106631870	4q24	NPNT, GSTCD	pass
**23**	rs13141641	145506456	4q31.21	HHIP	pass
**24**	rs13105210*	145262927	4q31.21	GYPA	pass
**25**	rs138641402*	145445779	4q31.21	HHIP-AS1	pass
**26**	rs9990514*	188842148	4q35.2	Intergenic	pass
**27**	rs7733088	147856333	5q32	HTR4	pass
**28**	rs10056066*	155861626	5q33.3	SGCD	MAF<0.01
**29**	rs2070600*	32151443	6p21.32	AGER	MAF<0.01
**30**	rs9394152*	33465482	6p21.32	ZBTB9	pass

**31**	rs3130559	31097301	6p21.33	PSORS1C1	pass
**32**	rs1265093	31107187	6p21.33	PSORS1C1, CCHCR1	pass
**33**	rs2074488	31240431	6p21.33	HLA-C	pass
**34**	rs9266629	31346822	6p21.33	FGFR3P	genotyped <80% samples
**35**	rs2076295	7563232	6p24.3	DSP	pass
**36**	rs2806356*	109266255	6q21	ARMC2	pass
**37**	rs9399401*	142668901	6q24.2	ADGRG6, GPR126	pass
**38**	rs858249	23220639	7p15.3	KLHL7, NUPL2	pass
**39**	rs78060357*	132056248	7q32.3	PLXNA4	pass
**40**	rs10491678	29568686	9p21.1	intergenic	pass
**41**	rs12245299*	55913700	10q21.1	PCDH15	pass
**42**	rs1923539	81694950	10q22.3	RP11-479O17.4	pass
**43**	rs7078012	81705433	10q22.3	SFTPD	pass
**44**	rs3923564	81735981	10q22.3	SFTPD	pass
**45**	rs12220777	81788104	10q22.3	RP11-369J21.6, RP11-369J21.5	pass
**46**	rs728616	81847914	10q22.3	RP11-369J21.2	pass
**47**	rs3851050	81882370	10q22.3	RP11-369J21.4	pass
**48**	rs207675*	79154537	10q22.3	KCNMA1	pass
**49**	rs721917*	81706324	10q22.3	SFTPD	pass
**50**	rs6585424*	81933748	10q22.3	ANXA11	pass
**51**	rs7079679*	102055546	10q24.31	PKD2L1	pass
**52**	rs4751240*	129136409	10q26.2	DOCK1	pass
**53**	rs7929679	34805849	11p13	APIP, EHF	pass
**54**	rs34391416	831818	11p15.5	EFCAB4A, CHID1, AP2A2	pass
**55**	rs2463822	62103420	11q12.3	ASRGL1	pass
**56**	rs3741240	62186542	11q12.3	SCGB1A1	pass
**57**	rs2077224	62197427	11q12.3	AHNAK	pass
**58**	rs17157266	62199817	11q12.3	AHNAK	pass
**59**	rs286499*	85193869	11q14.1	DLG2	pass
**60**	rs626750	102720945	11q22.2	MMP12	pass
**61**	rs16906714*	30860557	12p11.21	intergenic	Monomorphic
**62**	rs9599114*	66987131	13q21.32	PCDH9	pass
**63**	rs6576132	28728111	14q12	FOXG1	pass
**64**	rs140706189*	61151425	14q23.1	SIX1, SIX4, SIX6, MNAT1	pass
**65**	rs7140285	88462988	14q31.3	GALC	pass
**66**	rs754388	93115410	14q32.12	RIN3	pass
**67**	rs76677753*	34898066	15q14	GOLGA8B	Monomorphic
**68**	rs1441358*	71612514	15q23	THSD4	pass
**69**	rs7181486	78741618	15q25.1	IREB2, AGPHD1	pass
**70**	rs8042238	78774271	15q25.1	IREB2	pass
**71**	rs11858836	78783277	15q25.1	IREB2	pass
**72**	rs13180	78789488	15q25.1	CHRNA5, IREB2, CHRNA3	pass
**73**	rs8034191	78806023	15q25.1	CHRNA5, CHRNA3, CHRNA4, LOC123688	pass
**74**	rs17486278	78867482	15q25.1	CHRNA5, PSMA4, IREB2, AGPHD1, CHRNA3	pass
**75**	rs12914385	78898723	15q25.1	CHRNA3	pass
**76**	rs8192482*	78886198	15q25.1	CHRNA3	pass
**77**	rs8038108*	90089276	15q26.1	LINC00928, TICRR	pass
**78**	rs17707300*	28593347	16p11.2	CCDC101, IL27	pass
**79**	rs8050136	53816275	16q12.2	FTO	pass
**80**	rs7186831*	75473155	16q23.1	CFDP1	pass
**81**	rs114132812*	83817888	16q23.3	CDH13	Monomorphic
**82**	rs115067260*	83822302	16q23.3	CDH13	pass
**83**	rs145442019*	83844678	16q23.3	HSBP1	Monomorphic
**84**	rs8048576	84423034	16q24.1	ATP2C2	pass
**85**	rs2286351*	13928401	17p12	CDRT15P1	pass
**86**	rs140948272*	1460896	17p13.3	PITPNA	Monomorphic
**87**	rs647097*	8808464	18p11.22	MTCL1	pass
**88**	rs7937	41302706	19q13.2	RAB4B	pass
**89**	rs12459249*	41339896	19q13.2	CYP2A6	pass

* SNPs imputated

** Based on hg19 coordinates

Figure S2. Single-marker allelic association results.

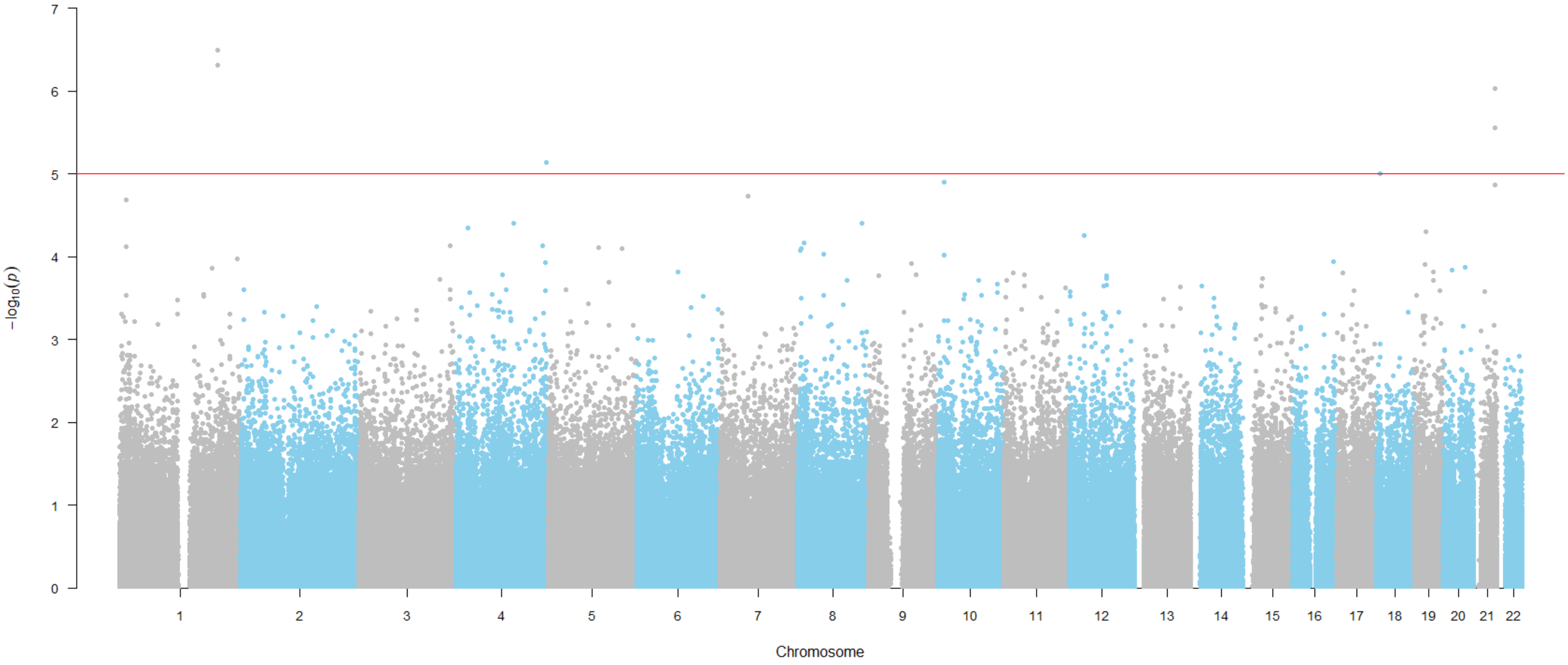

The Manhattan plot graphically represents the –log10 of the p value associated with COPD risk plotted across each chromosome based on 455,564 SNPs, after controlling for age, sex, the first two principal components of a PCA based on genetic data and the proportion of Mapuche ancestry (PMA)

Table S2. Single-SNP allelic association analysis (p-value<10^{-5}).

CHR	SNP	BP	Gene	Location	A1	OR	P
1	rs12741415	202474774	PPP1R12B	Non Coding Transcript Variant	A	3.01	3.18×10^{-7}
1	rs116062217	202431443	PPP1R12B	Intron	T	2.92	4.89×10^{-7}
21	rs1054761	43218520	PRDM15	Non Coding Transcript Variant	T	0.44	9.42×10^{-7}
21	rs4075967	43236176	PRDM15	Synonymous Variant	A	0.45	2.75×10^{-6}
4	rs4862451	185789995	Intergenic	-	G	2.47	7.21×10^{-6}

CHR: chromosome; SNP: single nucleotide polymorphism; BP: base pair; A1: minor allele frequency; OR: odds ratio; P: p-value.

Figure S3. Linkage disequilibrium plots around the *PRDM15* gene.

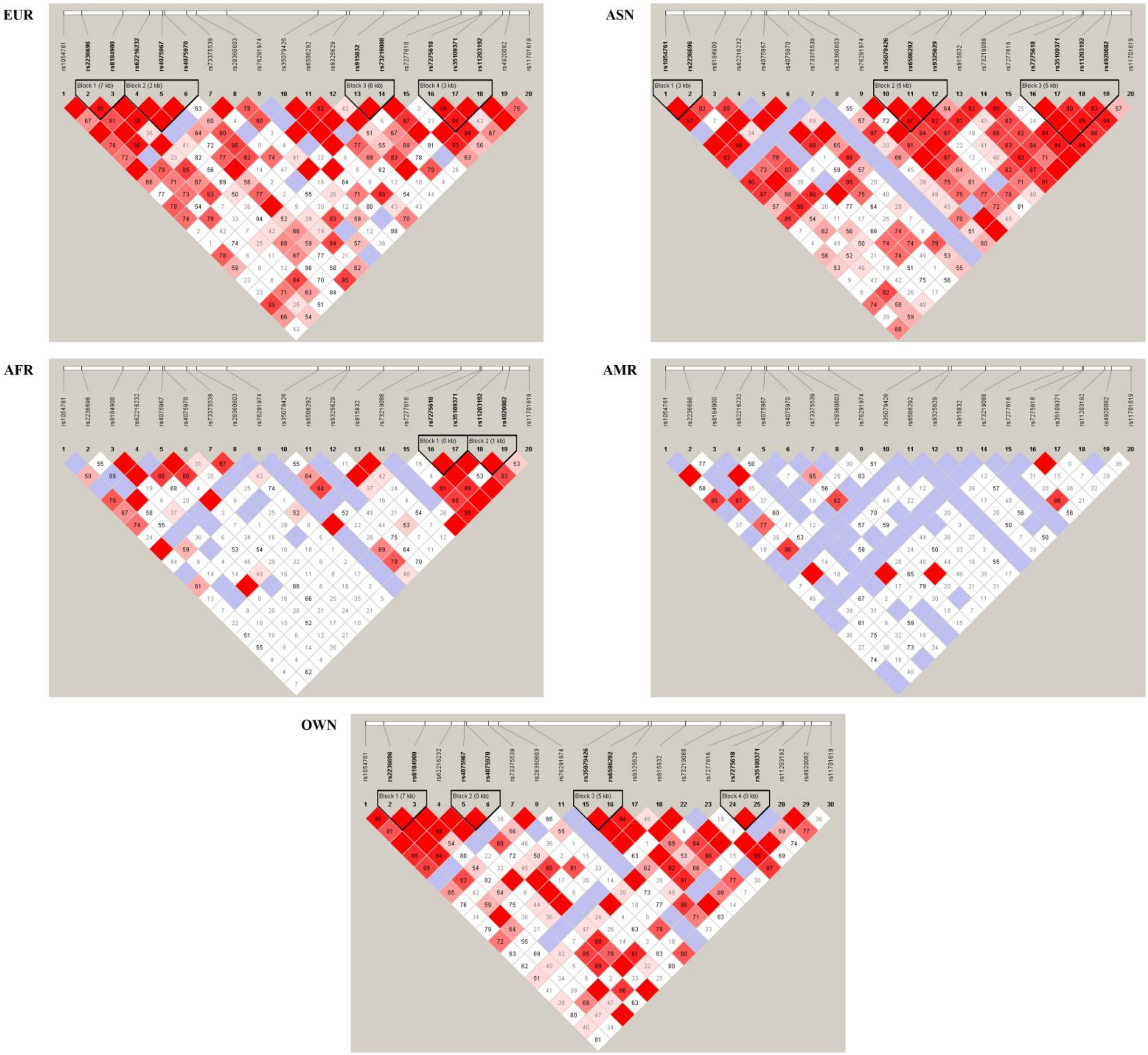

Squares represent the LD (r2) value between every 2 single-nucleotide polymorphisms: the more reddish the square, the higher the r2 pairwise value. Definition of abbreviations: AFR: Africans; AMR: admixed Americans; EAS: East Asians; EUR: Europeans; OWN: Our control population.
